# Determinants of regulatory compliance in health and social care services: a systematic review protocol

**DOI:** 10.12688/hrbopenres.13214.3

**Published:** 2021-06-16

**Authors:** Paul Dunbar, John P. Browne, Laura O'Connor

**Affiliations:** 1 Health Information and Quality Authority, Cork, T12 Y2XT, Ireland; 2 School of Public Health, University College Cork, Cork, Ireland

**Keywords:** Facility regulation and control, regulatory compliance, public policy, organisational culture, determinants

## Abstract

**Background: **The delivery of high quality health and social care services is a fundamental goal for health systems worldwide. Identifying the determinants of quality is a complex task as there are a myriad of variables to choose from. Researchers in this field have assessed a range of organisational and environmental factors (for example: staff composition, facility ownership, facility size) for an association with various quality metrics. Less attention has been paid to the determinants of compliance with quality regulation. Identifying the determinants of compliance has the potential to improve regulatory processes and can inform quality improvement initiatives undertaken by service providers and policy makers. This protocol describes a systematic review which will review literature from a wide range of study designs and sources to develop an overview of the determinants of regulatory compliance in health and social care services.

**Methods: **A wide range of study designs and grey literature will be sought for this review. Searches will be conducted using PubMed, MEDLINE, PsycInfo, SocINDEX and CINAHL databases. The studies included in the review will be subject to quality appraisal with reference to the collection of tools available from the Joanna Briggs Institute. Data extraction will be informed by the Consolidated Framework for Implementation Research (CFIR). A narrative synthesis will be conducted on the barriers, facilitators and factors associated with compliance, with reference to the concepts mapped onto the CFIR. GRADE-CERQual will be used to grade the overall body of evidence.

**Conclusion: **The findings of this review will be useful to regulators to inform regulatory policy and practice. Service providers and policy makers may also use the findings to inform quality improvement initiatives aimed at improving compliance and quality across a range of health and social care services.

## Introduction

The delivery of high quality health and social care services is a fundamental goal for health systems worldwide. Quality is a somewhat nebulous term that can be difficult to define. In health and social care, the following have been proffered — by the European Commission and the Institute of Medicine — as definitions of quality: “health care that is effective, safe and responds to the needs and preference of patients” ^1^; “Quality of care is the degree to which health services for individuals and populations increase the likelihood of desired health outcomes and are consistent with current professional knowledge” ^2^.

Quality of care is often conceptualised with reference to Donabedian’s framework ^3^: structure, process and outcome ^3^. Donabedian "defined structure as the environment in which healthcare is provided, process as the method by which healthcare is provided and outcome as the consequence of the healthcare provided" ^4^. To use social care as an example: the number of staff working in a centre would represent structure; the frequency with which a person has an assessment of need would represent process; and the degree to which a person has autonomy to make decisions about their care would be an outcome.

Quality of care in health and social care settings is variable. Variation in quality may be the result of a structural component as demonstrated in a study that assessed the effect of changes to staffing levels on the prevalence of pressure sores in nursing homes ^5^. It may be process-related as found in a prospective evaluation of simulated emergency department triage which found a high degree of variability in the processes of triage and measurement of vital signs ^6^. Outcomes are also subject to variation such as found in an analysis of in-hospital mortality in non-cardiac surgical patients across Europe, which found that mortality varied across the countries studied ^7^.

Regulation is one response to variability in quality: authorities establish a set of norms or standards to benchmark quality and then assess the extent to which organisations and individuals meet these standards. Failure to comply with regulations may lead to sanctions such as intensified surveillance, or even revocation of license to operate (typically through a registration or licensing system). Regulation is a common feature in a wide range of sectors: finance, environment, transport and healthcare. Selznick (1984) offers a useful definition of regulation where it is described as: “sustained and focused control exercised by a public agency over activities which are valued by a community” ^8^.

Regulations in health and social care are wide-ranging but typically cover aspects such as, hygiene, governance and management, documentation/records, care practices, staffing, training ^9– 12^. As with components of quality, regulations can also be conceived of as falling into the categories of structure, process and outcome. By way of example, in the context of staffing and patient experience: a regulation specifying the staffing ratio represents structure; a regulation specifying the supervision or development of staff in the humanity of care represents process; and a measure of patient experience represents the outcome.

Compliance, in a regulatory context, can be understood as “behavior fitting expectations communicated to regulatees regarding how the former should or should not behave in a given domain” ^13^. Compliance is generally articulated by a regulator along a continuum of ‘compliant’ to ‘not compliant’. For example, the Care Quality Commission (CQC), regulator for health and adult social care in England, has four levels of compliance: outstanding, good, requires improvement, inadequate ^14^. These ratings are determined according to the professional judgment of a CQC inspector and assessed at the level of individual care components; services also receive an overall rating.

Determining the level of compliance with a specific regulation requires varying degrees of effort and evidence-gathering on behalf of an inspector. Evidence can be generated from speaking with residents and staff, reviewing documentation and records, and observing practices as they happen. For example, assessing compliance with a structural requirement, such as a requirement that a person in charge should have “a minimum of 3 years’ experience in a management or supervisory role in the area of health or social care” ^15^, is a relatively straight-forward task of identifying the appropriate documentation. Judging whether a service has admitted residents in “a competent, equitable, timely, and respectful manner” ^16^, requires the inspector to undertake several tasks: speak with recently-admitted residents or their representatives, review admission records, speak with staff involved in admitting new residents. Assessing compliance in a regulation which specifies an outcome, such as one that seeks to “ensure respect for the personal privacy of each person in care” ^17^, requires the inspector to speak with residents and staff, review documentation and observe care practices on-site.

As with quality, compliance with regulations is also variable. This is evidenced in a number of reports by regulators in the health and social care sectors ^18– 20^. Various factors have been assessed for their association with compliance in health and social care settings such as: ownership, facility size, patient feedback, staff levels and competencies, availability of amenities, location, presence of an ombudsman and patient/resident characteristics ^21– 28^. In the case of some of the above, there is a danger of a type of circular reasoning because it may be a factor that is regulated. For example, if there is a regulation requiring managers to have a certain qualification, then it is difficult to construe this as a determinant because it is a pre-requisite for compliance.

Describing the reasons and potential explanations for the variability in compliance with regulations is the key focus for this systematic review. The authors have found no such review to date and this represents a gap in knowledge. Our review will use the Consolidated Framework for Implementation Research (CFIR) to categorise the determinants of compliance described in the literature.

The CFIR is an overarching typology used in implementation science ^29^. The CFIR was developed by including “constructs from a synthesis of existing theories” and is concerned more with what works where and why as opposed to simply ‘what works’ ^29^. There are five domains within the CFIR: intervention characteristics, outer setting, inner setting, characteristics of the individuals involved, and the process of implementation. Each of these have multiple constructs within each domain. 

The CFIR may be applied to regulation by mapping any barriers, facilitators or factors associated with levels of compliance. In this sense, regulation is perceived as the intervention and compliance is the outcome. By way of example, the points below illustrate how regulation and compliance can, hypothetically, be mapped onto the constructs within the five CFIR domains:

- intervention characteristics: do regulatees regard regulations as being evidence-based?
- outer setting: are there incentives/disincentives that encourage the regulatee to comply?
- inner setting: what resources (e.g. staffing) are available to the regulatee to achieve compliance?
- characteristics of the individuals: what knowledge and beliefs do senior managers in regulatees have towards the regulator?
- process of implementation: do inspectors act as external change agents to foster compliance?

Beyond mapping the barriers, facilitators or factors associated with levels of compliance our review will use theory to aid interpretation. It is not possible at this juncture to be definitive in what theories will be applicable, as this will be informed by the nature and design of the studies included for the systematic review. As such, the following sections will describe some potential theories that may serve to elucidate the material that is mapped on to the CFIR domains and constructs.

Various theories have been posited in the context of regulation and on the means by which regulators seek to ensure compliance ^30– 32^. The disposition or *modus operandi* of a regulator can be conceptualised as being plotted along a spectrum. At one end are those that are intolerant of any form of non-compliance and quick to deploy punitive measures. At the other end of the spectrum there is a greater acceptance that compliance can fluctuate and the regulator will adopt a more consultative approach which seeks to coax providers into compliance ^30– 32^.

Other theories look to characterise the disposition of regulatees and their attitude towards compliance ^31, 33, 34^. Non-compliant organisations may be ‘political citizens’, generally agreeing with the goals of regulation but objecting to the prescriptions of the regulator in terms of how this is to be achieved. Or, they may simply be ‘organisationally incompetent’ and fail to understand or manage the demands of the regulations or the regulator ^31^. Some organisations may fully subscribe to the goals of regulation and be ‘model citizens’ that strive to meet or exceed the standards that have been set. Others pay ‘lip service’ to these goals and do the minimum to satisfy the regulator, giving the appearance of compliance ^33^.

The regulator can also be interpreted by organisational actors as being either an ally, threat or obstacle ^35^. The regulator being perceived as a threat can mean the threat is at the level of an individual (their job or esteem) or at the level of the organisation (profits or reputation). As an ally, the regulator is perceived as competent and regarded as encouraging an organisation into compliance, offering advice and support to achieve common goals ^35^. The regulator as obstacle is seen as lacking authoritative technical expertise in the particular field or where “compliance requirements…are inadequately connected to the underlying regulatory goals” ^35^.

As set out above, compliance is not only influenced by structural or organisational factors such as the size of a facility or who owns it. It is also an outcome of the nature of engagement between regulator and regulatee and contingent on their respective dispositions towards compliance. Normalisation process theory (NPT) represents a theory within which to understand these relationships and contingencies. NPT facilitates “systematic exploration of why some processes lead to a practice becoming successfully (or not) embedded (i.e. normalised) and sustained, by attempting to understand the intervention in relation to the work that people do” ^36^. NPT facilitates an exploration of what factors are associated with the successful integration and alignment of an organisation’s goals with those of regulation. Such an approach has been adopted elsewhere ^36^ but the author has found no studies that have used NPT as a theory to aid understanding of regulatory compliance in organisations. NPT may be particularly useful in the fifth CFIR domain: process. 

The findings of this review will be of benefit to regulators as they may inform changes to regulatory policy and practice. In addition, service providers and policy makers may use the findings to develop quality improvement initiatives to improve rates of compliance and, ultimately, provide a better quality service.

**Research question:** What are the determinants of regulatory compliance in health and social care services?

The protocol will describe the:

- process for a comprehensive search for relevant articles
- eligibility criteria for the inclusion of such articles
- method for screening articles for inclusion
- appraisal method for assessing the quality of individual studies
- approach to data extraction, synthesis and appraisal of the overall body of evidence.

## Protocol

### Criteria for inclusion

The phenomena of interest are the determinants of regulatory compliance in health and social care services.

There are no limits on the articles for inclusion in terms of publication date or language.

Articles — either qualitative, quantitative or mixed-methods — will be included if they:

- Describe factors or characteristics that are related to regulatory compliance. Specifically, this refers to regulations that are mandated by government or other state authorities. A wide range of constructs will be considered for inclusion including, but not limited to, the following: service characteristics (size, location, model of care, ownership); organisational characteristics (culture, management/governance structure, maturity); service user characteristics (age, disability type, disease/illness); nature of engagement (punitive, adversarial, collaborative).
- Discuss barriers or facilitators to regulatory compliance for health and social care services.
- Are focused on quality of care in health and social care services and use regulatory compliance as an outcome measure.

Studies will be excluded if they:

- Analyse regulatory compliance in a field other than in a health or social care setting or service.
- Analyse compliance with clinical guidelines or other evidence-based methods for managing care that are not underpinned by the potential for regulatory sanction where there is a failure to comply.
- Use an outcome measure that is not equivalent to regulatory compliance in accordance with the definitions set out above. For example: adherence to voluntary standards or codes of conduct; where failure to comply does not result in regulatory sanctions of enforcement; compliance concerning individuals as opposed to organisations as is the case with regulations for specific health care professionals.

### Types of study to be included

There will be no specific limitations on the types of study considered for inclusion. Given the nature of the research question and the topic under review it is anticipated that the studies will generally fall into the categories of: cross-sectional designs for quantitative studies; and ethnographic or focus group/interview designs for qualitative studies. Preliminary searches have found studies that use compliance data from regulators coupled with cross-sectional data on services which are sourced either directly through surveys or via national repositories such as the Centers for Medicare and Medicaid Services (CMS) in the USA.

### Search methodology

The CIMO (Context, Intervention, Mechanisms, Outcome) framework for developing a search strategy will be used for this systematic review (see Table 1). The search terms in the framework are set out in Table 2 below.

**Table 1.** Context, Intervention, Mechanisms, Outcome (CIMO) framework for this study.

**C**ontext
Any health or social care service (e.g. hospital, nursing home, residential disability service) which is regulated as an organisation. This excludes services that are provided by individual professionals such as dentists or general practitioners.
**I**ntervention(s)
Regulation is the intervention. The term ‘regulation’ may differ in a given context but it is generally taken to mean a process of external evaluation by an independent or statutory agency which is underpinned by enforcement powers.
**M**echanism
Factors influencing, or determinants of, compliance; barriers and facilitators to compliance; nature of engagement between regulator and regulatee.
**O**utcome
**Main outcome:** regulatory compliance. **Additional outcome(s):** Other measures that are consistent with compliance will be included (e.g. in the USA, equivalent terms for non-compliance may be a ‘deficiency’ or ‘violation’).

**Table 2.** Key search terms.

Context	“healthcare system*” OR “health care system*” OR “care system*” OR “social care” OR ”healthcare service*” OR ”health care service*” OR ”social care service*” OR “hospital*” OR “health care setting*” OR “healthcare setting*” OR “social care setting” OR “residential facilit*” OR “care facility*” OR “nursing home*” OR “residential care” OR “long-term care” OR “long term care” OR “disabilit*” OR “disability service” OR “care home” OR “aged care” OR “aged-care” OR “mental health service” OR “mental health centre” OR “mental health facilit*” OR “psychiatric service” OR “psychiatric centre” OR “psychiatric facilit*” OR “addiction service” OR “addiction centre” OR “addiction facilit*” OR “drug- treatment centre” OR “drug-treatment service” OR “drug-treatment facilit*” OR “drug treatment centre” OR “drug treatment service” OR “drug-treatment facilit*” OR “homecare” OR “home care” OR “domiciliary” OR “primary care” OR “community care” OR “respite care” OR “specialist care” OR “live-in care” OR “live in care” OR “homeless service*” OR “homeless shelter*”
Intervention	“regulation” OR “regulator*” OR “inspect*” OR “enforcement” OR “licens*” OR “certification” OR “withdrawal”
Mechanisms	“factor*” OR “barrier*” OR “facilitator*” OR “enabler*” OR “determinant*” OR “characteristic*” OR “indicator*” OR “association*” OR “relationship*” OR “cause*” OR “engagement” OR “attitude*” OR “predictor*”
Outcome	“compliance” OR “non-compliance” OR “violat*” OR “deficienc*” OR “sanction*” OR “citation*” OR “failure*” OR “failing*”

### Information sources

Searches will be carried out on the following databases: PubMed, MEDLINE, PsycINFO, CINAHL and SocINDEX. In addition, the bibliographies of the included full-text articles will be hand searched for relevant articles. Forward citation searching will also be carried out to identify other potential material for inclusion. The search terms for one electronic database (PubMed) are provided in Table 2.

Searches will also be conducted on established grey literature databases including OpenGrey System for Information on Grey Literature in Europe and OpenSIGLE. Targeted searches will also be carried out on websites of regulatory organisations and Government agencies/departments involved in health and social care regulation, identified by referring to the Organisation for Economic Development and Cooperation’s (OECD) resources on regulatory policy internationally ^37^.

### Software

The software used for screening articles is the online tool Covidence and the bibliography manager is EndNote X8.2 by PDF Tron™ Systems Inc.

### Screening

All references returned by the search terms from each information source will be imported into Covidence. Duplicate references will be removed. Two researchers will independently screen the titles and abstracts of each of the retrieved articles against the inclusion/exclusion criteria using Covidence. Any disagreements on inclusion/exclusion will be resolved, in the first instance, by discussion. Any disagreements not resolved by discussion will be adjudicated on by a third author. Full-text review and bibliography searches will be performed by PD. The search strategy and study selection process will be reported in accordance with the Preferred Reporting Items for Systematic reviews and Meta-Analyses (PRISMA) statement ^38^. A PRISMA flow diagram will be generated.

### Quality appraisal

As referenced above, a wide range of study designs will be considered for inclusion in the systematic review. Preliminary searches have found studies that are entirely quantitative or qualitative as well as mixed-methods. Quality appraisal tools will be selected dependent on the type of study. As such, the suite of appraisal tools made available by the Joanna Briggs Institute will be used ^39^. Grey literature will be appraised for quality with reference to Tyndall’s checklist which assesses the following aspects: authority, accuracy, coverage, objectivity, date and significance (AACODS) ^40^.

Two reviewers will independently assess the quality of articles selected for data extraction. Any disagreements on quality appraisal will be resolved by consensus or, if necessary, a third author will be consulted for final decision. 

### Data extraction

Two reviewers will independently carry out data extraction of all articles deemed eligible for inclusion, using a data extraction table (see *Extended data* ^41^). The data to be extracted includes general information (title, author(s), publication date) as well as specific data under each of the CFIR domains. The data extraction template has been piloted and refined using studies retrieved during preliminary searches. The extracted data will be compared to ensure agreement and identify any discrepancies; disagreements will be resolved by discussion. Any disagreements not resolved by discussion will be referred to a third author for arbitration or, where appropriate, through contact with the study authors. 

### Data synthesis

Due to the wide range and heterogeneity of the studies that will be returned through the search strategy, a narrative synthesis will be performed. Narrative synthesis uses text and illustrations to describe, compare and combine heterogeneous qualitative findings and quantitative results ^42^. This approach places the focus on the interpretive synthesis of the narrative aspects of research findings, as opposed to any attempt to synthesise findings in a quantitative manner, such as in a meta-analysis.

The extracted data will first be tabulated in accordance with the CFIR and its respective domains and constructs. The data will then be summarised in narrative form, incorporating elements of *Popay et al.*’s ^43^ methodology of narrative synthesis, and in line with the CFIR domains and constructs in addition. Overall confidence in the evidence will be appraised with reference to GRADE-CERQual ^44^.

### Dissemination of information

The systematic review will be submitted to an academic journal on completion. Conference abstracts arising out of the systematic review will also be submitted to appropriate conferences for presentation. The findings of the systematic review will be circulated and presented to regulation staff in the Health Information and Quality Authority, Ireland. Review findings will be circulated to other regulators in Europe through the Supervision and regulation Innovation Network for Care (SINC).

### Study status

Database searches using the search terms outlined in Table 2 have commenced.

### Strengths and limitations

To the best of the author’s knowledge, this review will be the first to systematically assess the determinants of regulatory compliance. In addition, the methodological approach (including a wide range of study designs; synthesising the data using narrative synthesis with reference to the CFIR) allows for a comprehensive exploration of what factors are associated with the successful integration of regulatory requirements with an organisation’s goals. The use of GRADE-CERQual in appraising the quality of the overall body of evidence will aid knowledge users in establishing which determinants are appropriate for inclusion in any quality improvement initiatives.

In terms of limitations, it is possible that some relevant studies may not be retrieved due to the multiplicity of terms used in the literature to refer to regulation and compliance. This has been ameliorated with reference to resources from a range of countries to ensure that equivalent words and phrases are used in the search terms.

## Conclusion

This protocol describes the methodological approach for searching, synthesising and quality appraisal of the available literature to answer the research question, what are the determinants of regulatory compliance in health and social care services? The findings of the systematic review will be of interest to organisations working in a regulatory capacity across diverse fields and may also inform quality improvement initiatives for service providers and policy makers in the health and social care sector.

## Data availability

### Underlying data

No data are associated with this article.

### Extended data

Figshare: Supplementary File 1 - Data Extraction Tool.docx https://doi.org/10.6084/m9.figshare.13546664 ^41^

This project contains the following extended data:

- Supplementary file 1. This data extraction tool provides for the extraction of data for the systematic review. It includes general information about studies in addition to fields specific to the Consolidated Framework for Implementation Research (CFIR).

### Reporting guidelines

Figshare: PRISMA-P checklist for ‘Determinants of regulatory compliance in health and social care services: a systematic review protocol’ https://doi.org/10.6084/m9.figshare.13553882.v2 ^45^

Data are available under the terms of the Creative Commons Attribution 4.0 International license (CC-BY 4.0).
