# Supplementary material for: Determinants of regulatory compliance in health and social care services: a systematic review protocol
Source: HRB Open Res. 2021 Jun 16;4:13. Originally published 2021 Jan 28. [Version 3] doi: 10.12688/hrbopenres.13214.3 (PMC8258703; doi:10.12688/hrbopenres.13214.3)
Supplement: Peer review history

# Peer review: Reviewer response for version 3

**DOI:** 10.21956/hrbopenres.14520.r29683

**Published:** 2021-07-05

**Authors:** Xiaochuan Wang

**Affiliations:** 1 School of Social Work, College of Health Professions and Sciences, University of Central Florida, Orlando, FL, USA

I’ve reviewed the revised article. I appreciate the efforts that the authors put to address the reviewer’s suggestions and feedback. I have no further comments.

Is the study design appropriate for the research question?

Yes

Is the rationale for, and objectives of, the study clearly described?

Yes

Are sufficient details of the methods provided to allow replication by others?

Yes

Are the datasets clearly presented in a useable and accessible format?

Yes

Reviewer Expertise:

NA

I confirm that I have read this submission and believe that I have an appropriate level of expertise to confirm that it is of an acceptable scientific standard.

# Peer review: Reviewer response for version 2

**DOI:** 10.21956/hrbopenres.14432.r29203

**Published:** 2021-06-01

**Authors:** Xiaochuan Wang

**Affiliations:** 1 School of Social Work, College of Health Professions and Sciences, University of Central Florida, Orlando, FL, USA

Thank you for the opportunity to review this protocol. This protocol describes a systematic review that aims to provide an overview of determinants of compliance with quality regulation in health and social care services. The focus of the proposed study represents an understudied and important topic. As evidenced in the current version, a number of changes have been made to strengthen the protocol. I have some minor comments that may help to further improve the paper:

**Introduction:**

 p.3, paragraph 2- I suggest the authors give some more details about the Donabedian's framework (e.g., what do the three domains- structure, process, outcome- refer to), instead of just providing an example for each.

 p.3, paragraph 5- Need citation for “Regulations in health and social care are wide-ranging but …training.”

**Theoretical framework:**

 p.4- The inclusion of CFIR framework and potential theories is appreciated as it helped to improve clarity. However, I believe the theoretical framework section can be developed more clearly and in more details. For example, I am wondering if the question listed in each of the five domains is comprehensive enough. Take outer setting domain as an example, you currently list “are there incentives/disincentives that encourage the regulatee to comply” as the question. But how about contextual level factors that could also affect regulation yet not necessarily providing incentives/disincentives? Please at least clarify whether these questions are exhaustive or are provided just as examples.

**Table 2:**

 p.6- Consider add “predictor*” to “Mechanisms”.

Is the study design appropriate for the research question?

Yes

Is the rationale for, and objectives of, the study clearly described?

Yes

Are sufficient details of the methods provided to allow replication by others?

Yes

Are the datasets clearly presented in a useable and accessible format?

Yes

Reviewer Expertise:

NA

I confirm that I have read this submission and believe that I have an appropriate level of expertise to confirm that it is of an acceptable scientific standard, however I have significant reservations, as outlined above.

# Response

**Published:** 2021-06-11

**Authors:** Paul Dunbar

**Affiliations:** Hiqa, Ireland

We would like to thank Ms Wang for her valuable additional suggestions. Revisions have been made and are individually detailed below. We believe the revisions, informed by your comments, enhance the systematic review protocol greatly.

- *Italic text*: reviewer’s comments.
- **Bold Text:** response from the Authors

*p.3, paragraph 2- I suggest the authors give some more details about the Donabedian's framework (e.g., what do the three domains- structure, process, outcome- refer to), instead of just providing an example for each.*

**We thank the reviewer for drawing attention to this oversight. The text has been amended to include a quotation and reference which we consider addresses the reviewer’s comment. See below:**

**Donabedian "defined structure as the environment in which healthcare is provided, process as the method by which healthcare is provided and outcome as the consequence of the healthcare provided".**

**Reference: **Rademakers J, Delnoij D, de Boer D. Structure, process or outcome: which contributes most to patients' overall assessment of healthcare quality?. BMJ quality & safety. 2011 Apr 1;20(4):326-31.

*p.3, paragraph 5- Need citation for “Regulations in health and social care are wide-ranging but …training.”*

**We thank the reviewer for drawing attention to the need to provide a reference for this statement. To address this, the sentence is now followed by citations to four documents which outline regulations in various jurisdictions and settings. See references below: **

1. **Government of Ireland. Care and Welfare of Residents in Designated Centres for Older People Regulations 2013. Dublin, The Stationery Office; 2013. Available from: http://www.irishstatutebook.ie/eli/2013/si/415/made/en/print**
2. **Welsh Government. Statutory Guidance - For service providers and responsible individuals on meeting service standard regulations. Wales, Welsh Government; 2019. Available from: https://gov.wales/sites/default/files/publications/2019-04/guidance-for-providers-and-responsible-individuals.pdf**
3. **Government of New Zealand. Health and Disability Services (Safety) Act 2001. Wellington, New Zealand: Government of New Zealand; 2001.**
4. **Government of British Columbia. Community Care and Assisted Living Regulation. Victoria, British Columbia: Queen's Printer; 2004.**

*p.4- The inclusion of CFIR framework and potential theories is appreciated as it helped to improve clarity.* *^(2)^* *However, I believe the theoretical framework section can be developed more clearly and in more details. For example, I am wondering if the question listed in each of the five domains is comprehensive enough. Take outer setting domain as an example, you currently list “are there incentives/disincentives that encourage the regulatee to comply” as the question. But how about contextual level factors that could also affect regulation yet not necessarily providing incentives/disincentives? Please at least clarify whether these questions are exhaustive or are provided just as examples.*

**Thank you for the opportunity to clarify this important section of the protocol. The questions referred to by the reviewer are only intended for illustrative purposes. This is stated in the preceding sentence where it is noted that they are included by way of example. The preceding sentence is as follows:** **“** **By way of example, the points below illustrate how regulation and compliance can, hypothetically, be mapped onto the constructs within the five CFIR domains:”.**

**As such, it is our intention to leave this section unchanged.**

p.6- Consider add “predictor*” to “Mechanisms”.

**This suggestion is welcome and we agree that this word should be added to the search terms in order to generate a more comprehensive list of results. Table 2 has now been revised to include this term.**

# Peer review: Reviewer response for version 2

**DOI:** 10.21956/hrbopenres.14432.r29198

**Published:** 2021-03-26

**Authors:** Alan Boyd

**Affiliations:** 1 Alliance Manchester Business School, University of Manchester, Manchester, UK

I would like to thank the authors for their comprehensive and considered responses to my original comments. I have no further comments to make.

Is the study design appropriate for the research question?

Yes

Is the rationale for, and objectives of, the study clearly described?

Yes

Are sufficient details of the methods provided to allow replication by others?

Yes

Are the datasets clearly presented in a useable and accessible format?

Yes

Reviewer Expertise:

Regulation of health and social care services.

I confirm that I have read this submission and believe that I have an appropriate level of expertise to confirm that it is of an acceptable scientific standard.

# Peer review: Reviewer response for version 1

**DOI:** 10.21956/hrbopenres.14366.r28908

**Published:** 2021-03-03

**Authors:** Alan Boyd

**Affiliations:** 1 Alliance Manchester Business School, University of Manchester, Manchester, UK

I think this could be a really insightful review, although tricky to conceptualise and operationalise. The idea of using Normalisation Process Theory (NPT) and the Consolidated Framework for Implementation Research (CFIR) is good. I wish I had thought of it!

 However, I am not totally clear from this protocol as to how NPT and CFIR fit together. I am not sure whether it is just a case of needing to make things a bit clearer for readers, or whether there is something more fundamental. I can see NPT as relating particularly to implementation processes, but it is not clear to me exactly how NPT facilitates understanding of other CFIR categories. Another example or two might be instructive? Ideally perhaps you might include a table to show how different elements of NPT and CFIR complement each other?

 Related to this, in your Data Extraction and Data Synthesis sections I can't see any mention of NPT, only of CFIR. Shouldn't NPT also be part of your data extraction and analysis?

 I also found your bullet points about how CFIR may be applied to regulation and compliance difficult to understand. I think there could be alternative ways of mapping regulation and compliance onto CFIR, so perhaps some further explanation would be helpful? For example, is the process of implementation more than this? E.g. sharing of / publicising standards? You have separated out the regulator as the only actor involved in implementation. But what about, for example, the regulated organisation incorporating those standards into its own QA or QI reporting or processes? You may have put this under Inner Context, but why?

 One further thought is whether/where patients/service users (and perhaps other stakeholders) appear, in view of "tripartite" regulation? Perhaps they are part of the outer setting?

 I also have a few small suggestions regarding the practical details of the protocol.

 Excluding voluntary standards or codes of conduct may be a grey area, in that some "voluntary" accreditations may be mandated by ministries of health and hence might be viewed as regulation.

 I wonder if there is something missing from the exclusion/inclusion criteria? By "regulatory compliance" you don't just mean compliance with standards subscribed to by regulators, but also the process of regulation (inspection frequencies, nature, sanctions etc.) - as per your "implementation process" category above. However, as I understand your inclusion criteria, they could apply to E.g. NICE guidelines that regulators also assess against. There will be lots of papers that assess implementation/impact of NICE guidelines without any reference to regulator use of those guidelines. If these are included then the scope of the review could be very large (and difficult to determine - you would need to know which guidelines regulators refer to). So perhaps you need something more explicit in your criteria about active regulation being part of the context which included research studies consider? Reading on I see that perhaps you have this covered under the Intervention part of the CIMO framework for the study, but it doesn't come across in the description in the main text of the study protocol.

 Table 2: Key search terms

 You might perhaps consider adding:

 Context: primary care; home / domiciliary care

 Intervention: certification

 Mechanisms: withdrawal (of licence)

 Are you going to do any citation searching in addition to keyword searching? I think the Mechanism element of CIMO is tricky for this search, and you might miss some research as a result.

 Finally, I have some small points about some of the wording in the Introduction section (although they might possibly indicate a need to be a bit clearer conceptually?):

 Paragraph 3. I did not find the process example particularly illuminating, and wonder if you can find something more obviously related to process. I appreciate that the boundaries between structure and process can be fuzzy, though. The structure and process examples relate these to outcomes. The outcomes example relates outcomes to countries. It might perhaps be better to put this example first, and then to put the others which relate outcome variation to structure and process?

 Paragraph 4. "typically fall under" is a bit ambiguous. The structure, process, outcome framework could be applied to any set of standards; it is an empirical question whether governments organise their standards in this way (explicitly or implicitly). This would be strengthened by a supporting reference. If this isn't available, perhaps add an example or two to help back this up?

 Paragraph 4. "must then comply with regulations...." Strictly speaking, be assessed as complying with regulations, and avoid potential sanctions or other potential negative consequences? Think, for example of reputation (as you highlight later) and "sunshine regulation".

 Column 2, Paragraph 1: CQC as an example of compliant/non-compliant continuum works better with regard to the continuum aspect than the compliant/non-compliant aspect, although CQC assessment does in some ways equate Good with compliant and Requires Improvement with non-compliant. There is also the point that CQC assessments are based on professional judgement and not purely on legal standards, which may complicate things?

 Column 2, Paragraph 2: "requires a little more interrogation and evidence gathering". And also a value judgement, although measures of competence etc. might possibly be set out?

 Column 2, Paragraph 2: "requires a lot of evidence gathering as well as observation". I don't see how this example is different to admitting in a respectful manner?

 Also a point perhaps that observation is one way of generating evidence? By evidence gathering perhaps you mean documentary evidence? Could be clearer?

Is the study design appropriate for the research question?

Yes

Is the rationale for, and objectives of, the study clearly described?

Yes

Are sufficient details of the methods provided to allow replication by others?

Yes

Are the datasets clearly presented in a useable and accessible format?

Yes

Reviewer Expertise:

Regulation of health and social care services.

I confirm that I have read this submission and believe that I have an appropriate level of expertise to confirm that it is of an acceptable scientific standard, however I have significant reservations, as outlined above.

# Response

**Published:** 2021-03-16

**Authors:** Paul Dunbar

**Affiliations:** Hiqa, Ireland

***Responses to reviewer Mr Alan Boyd’s feedback on the manuscript submitted by Dunbar et al., “ Determinants of regulatory compliance in health and social care services: a systematic review protocol” (https://doi.org/10.12688/hrbopenres.13214.1)***

 We would like to thank Mr Boyd for his valuable and constructive comments. Revisions have been made and are individually detailed below. We believe the revisions, informed by your comments, enhance the systematic review protocol greatly. The principal amendment is a reconfiguration of the section outlining the theoretical framework for the systematic review.  Any minor revisions highlighted in-text and not explained were made in accordance with or as a result of the changes outlined in this document.

- *Italic text*: reviewer’s comments.
- **Bold Text:** response from the Authors

*I. I am not totally clear from this protocol as to how NPT and CFIR fit together. I am not sure whether it is just a case of needing to make things a bit clearer for readers, or whether there is something more fundamental. I can see NPT as relating particularly to implementation processes, but it is not clear to me exactly how NPT facilitates understanding of other CFIR categories. Another example or two might be instructive? Ideally perhaps you might include a table to show how different elements of NPT and CFIR complement each other?*

*Related to this, in your Data Extraction and Data Synthesis sections I can't see any mention of NPT, only of CFIR. Shouldn't NPT also be part of your data extraction and analysis?*

**We thank the reviewer for drawing attention to this important element of the protocol. We agree that the manner of interaction between the CFIR and NPT could have been explained more clearly and this feedback has aided our thinking in this regard. In response, we have restructured the section in the main body of the text that sets out the theoretical framework. The use of the CFIR as a mapping device for the identification of enablers and barriers, has been introduced earlier in the section and the theory now comes later. In addition, the text makes clearer that NPT is one of several potential theoretical frameworks which can aid understanding and contextualising the enablers and barriers mapped in the CFIR. The other theories discussed in the main text around how regulators and regulatees behave and interact with one another are now included alongside NPT as several possible theories to help interpret the study’s findings.**

**In addition to these edits, two other references to the use of the CFIR in conjunction with NPT (in the abstract and in the strengths/limitations sections) have been edited to remove reference to the NPT and leave open the possibility of using several theories, including NPT to aid explanation of the findings.**

**As to the comment regarding the data extraction and data synthesis, we believe the above response helps explain why NPT is not included in any of the data extraction methodology. Moreover, the choice of theory to aid data synthesis is not yet determined. It may draw on one of the several theories outlined in the main body of the protocol or rely on other theories that are identified in the literature. The choice of theories will be dependent on the enablers and barriers identified during the review.**

**The new section outlining the theory is set out below:**

**“** **Describing the reasons and potential explanations for the variability in compliance with regulations is the key focus for this systematic review. The authors have found no such review to date and this represents a gap in knowledge. Our review will use the Consolidated Framework for Implementation Research (CFIR) to categorise the determinants of compliance described in the literature.**

**The CFIR is an overarching typology used in implementation science ^31^ . The CFIR was developed by including “constructs from a synthesis of existing theories” and is concerned more with what works where and why as opposed to simply ‘what works’ ^31^ . There are five domains within the CFIR: intervention characteristics, outer setting, inner setting, characteristics of the individuals involved, and the process of implementation. Each of these have multiple constructs within each domain.**

**The CFIR may be applied to regulation by mapping any barriers, facilitators or factors associated with levels of compliance. In this sense, regulation is perceived as the intervention and compliance is the outcome. By way of example, the points below illustrate how regulation and compliance can, hypothetically, be mapped onto the constructs within the five CFIR domains:**

- **intervention characteristics: do regulatees regard regulations as being evidence-based?**
- **outer setting: are there incentives/disincentives that encourage the regulatee to comply?**
- **inner setting: what resources (e.g. staffing) are available to the regulatee to achieve compliance?**
- **characteristics of the individuals: what knowledge and beliefs do senior managers in regulatees have towards the regulator?**
- **process of implementation: do inspectors act as external change agents to foster compliance?**

**Beyond mapping the barriers, facilitators or factors associated with levels of compliance our review will use theory to aid interpretation. It is not possible at this juncture to be definitive in what theories will be applicable, as this will be informed by the nature and design of the studies included for the systematic review. As such, the following sections will describe some potential theories that may serve to elucidate the material that is mapped on to the CFIR domains and constructs.**

**Various theories have been posited in the context of regulation and on the means by which regulators seek to ensure compliance ^24^^– ^^26^ . The disposition or *modus operandi* of a regulator can be conceptualised as being plotted along a spectrum. At one end are those that are intolerant of any form of non-compliance and quick to deploy punitive measures. At the other end of the spectrum there is a greater acceptance that compliance can fluctuate and the regulator will adopt a more consultative approach which coaxes providers into compliance ^24^^– ^^26^ .**

**Some studies have analysed the disposition of regulatees and their attitude towards compliance ^25^^, ^^27^^, ^^28^ . Non-compliant organisations may be ‘political citizens’, generally agreeing with the goals of regulation but objecting to the prescriptions of the regulator in terms of how this is to be achieved. Or, they may simply be ‘organisationally incompetent’ and fail to understand or manage the demands of the regulations or the regulator ^25^ . Some organisations may fully subscribe to the goals of regulation and be ‘model citizens’ that strive to meet or exceed the standards that have been set. Others pay ‘lip service’ to these goals and do the minimum to satisfy the regulator, giving the appearance of compliance ^27^ .**

**The regulator can also be interpreted by organisational actors as being either an ally, threat or obstacle ^29^ . The regulator being perceived as a threat can mean the threat is at the level of an individual (their job or esteem) or at the level of the organisation (profits or reputation). As an ally, the regulator is perceived as competent and regarded as encouraging an organisation into compliance, offering advice and support to achieve common goals ^29^ . The regulator as obstacle is seen as lacking authoritative technical expertise in the particular field or where “compliance requirements…are inadequately connected to the underlying regulatory goals” ^29^ .**

**As set out above, compliance is not only influenced by structural or organisational factors such as the size of a facility or who owns it. It is also an outcome of the nature of engagement between regulator and regulatee and contingent on their respective dispositions towards compliance. Normalisation process theory (NPT) represents a theory within which to understand these relationships and contingencies. NPT facilitates “systematic exploration of why some processes lead to a practice becoming successfully (or not) embedded (i.e. normalised) and sustained, by attempting to understand the intervention in relation to the work that people do” ^30^ . NPT facilitates an exploration of what factors are associated with the successful integration and alignment of an organisation’s goals with those of regulation. Such an approach has been adopted elsewhere ^30^ but the author has found no studies that have used NPT as a theory to aid understanding of regulatory compliance in organisations. NPT may be particularly useful in the fifth CFIR domain: process.”**

*II. I also found your bullet points about how CFIR may be applied to regulation and compliance difficult to understand. I think there could be alternative ways of mapping regulation and compliance onto CFIR, so perhaps some further explanation would be helpful? For example, is the process of implementation more than this? E.g. sharing of / publicising standards? You have separated out the regulator as the only actor involved in implementation. But what about, for example, the regulated organisation incorporating those standards into its own QA or QI reporting or processes? You may have put this under Inner Context, but why?*

**We thank the reviewer for identifying the improvement opportunity in which the bullet points seek to show how CFIR might be applied to regulation and compliance. We have amended the text to use new examples and make specific reference to the terminology used within each of the CFIR domains. The revised introductory text and bullet points are as follows:**

**“The CFIR may be applied to regulation by mapping any barriers, facilitators or factors associated with levels of compliance. In this sense, regulation is perceived as the intervention and compliance is the outcome. By way of example, the points below illustrate how regulation and compliance can, hypothetically, be mapped onto the constructs within the five CFIR domains:**

- **intervention characteristics: do regulatees regard regulations as being evidence-based?; what cost is incurred by the regulatee in complying?**
- **outer setting: what are the demographics of those whose needs are served by the regulatee?; are there incentives/disincentives that encourage the regulatee to comply?**
- **inner setting: what resources (e.g. staffing) are available to the regulatee to achieve compliance?; is the culture within the organisation positive towards the regulator?**
- **characteristics of the individuals: what personal attributes do individual inspectors exhibit when confronted with non-compliance?; what knowledge and beliefs do senior managers in regulatees have towards the regulator?**
- **process of implementation: are there specific staff appointed to champion regulatory compliance within regulatees?; do inspectors act as external change agents to foster compliance?”**

**Furthermore, we agree with the reviewer’s point on the regulator being the only actor involved in implementation. We have, therefore, included reference to the regulatee and to service users in the examples above.**

*III. One further thought is whether/where patients/service users (and perhaps other stakeholders) appear, in view of "tripartite" regulation? Perhaps they are part of the outer setting?*

**The reviewer raises a legitimate point on the inclusion of patients and service users and where they might feature in the context of the CFIR. We agree that there should be reference to service users and their potential to influence the level of regulatory compliance. The section of the protocol outlining how regulation and compliance can be mapped onto CFIR (see revised bullet points in II. above) now includes an example of how the demographics of the service users can impact on the ability to comply.**

*IV. Excluding voluntary standards or codes of conduct may be a grey area, in that some "voluntary" accreditations may be mandated by ministries of health and hence might be viewed as regulation.*

**The reviewer raises an important distinction regarding the grey area of accreditation, codes of conduct and voluntary standards. We agree that this requires further explanation in order to clarify the distinction between what is ‘regulated’ and what is ‘monitored’ or aspired to as best practice. As such, we have included a sentence in the exclusion criteria to explicitly state that voluntary standards/codes will excluded where they are not backed up by regulatory sanction. The revised text is set out below:**

**“Specifically, this refers to regulations that are mandated by government or other state authorities.”**

*V. I wonder if there is something missing from the exclusion/inclusion criteria? By "regulatory compliance" you don't just mean compliance with standards subscribed to by regulators, but also the process of regulation (inspection frequencies, nature, sanctions etc.) - as per your "implementation process" category above. However, as I understand your inclusion criteria, they could apply to E.g. NICE guidelines that regulators also assess against. There will be lots of papers that assess implementation/impact of NICE guidelines without any reference to regulator use of those guidelines. If these are included then the scope of the review could be very large (and difficult to determine - you would need to know which guidelines regulators refer to). So perhaps you need something more explicit in your criteria about active regulation being part of the context which included research studies consider? Reading on I see that perhaps you have this covered under the Intervention part of the CIMO framework for the study, but it doesn't come across in the description in the main text of the study protocol.*

**We thank the reviewer for identifying that regulatory compliance can encompass many considerations and guidelines that complement regulation. We agree that this should be addressed to clearly state what is meant by ‘regulatory compliance’. We have addressed this in the inclusion/exclusion criteria by being explicit about what ‘regulatory compliance’ means and also excluding any studies which focus on compliance with clinical guidelines that are not supported by regulatory enforcement. The revised text is underlined below:**

**“Studies will be excluded if they:**

- **Analyse regulatory compliance in a field other than in a health or social care setting or service. **
- **Analyse compliance with clinical guidelines or other evidence-based methods for managing care that are not underpinned by the potential for regulatory sanction where there is a failure to comply.**
- **Use an outcome measure that is not equivalent to regulatory compliance in accordance with the definitions set out above. For example: adherence to voluntary standards or codes of conduct where failure to comply does not result in regulatory sanctions of enforcement; compliance concerning individuals as opposed to organisations as is the case with regulations for specific health care professionals.”**

VI. *Table 2: Key search terms* *You might perhaps consider adding:*

*Context: primary care; home / domiciliary care*

*Intervention: certification*

*Mechanisms: withdrawal (of licence)*

**We thank the reviewer for the suggested additional search terms. On reflection, we agree that the terms require expanding to capture a wider range of health and social care services. In terms of the ‘Context’ field above, we have included the terms primary, home and domiciliary care as suggested. In addition, we have decided to include further terms related to the following services which would commonly be subject to regulation: mental health, addiction, homeless services, respite, community care and specialist care.**

**In addition, we agree that the word certification merits inclusion in the ‘Intervention’ field and have done so. Finally, under ‘Mechanism’, we disagree that the term ‘withdrawal’ should be included here. The terms included in this field relate to phenomena that may promote, inhibit or be in some way associated with compliance (for example, ‘barriers’, ‘indicators’, ‘cause’). The term ‘withdrawal’ is more applicable as a regulatory intervention. As such, we have decided to include it in the intervention field.**

**Context**

“healthcare system*” OR “health care system*” OR “care system*” OR “social care” OR ”healthcare service*” OR ”health care service*” OR ”social care service*” OR “hospital*” OR “health care setting*” OR “healthcare setting*” OR “social care setting” OR “residential facilit*” OR “care facility*” OR “nursing home*” OR “residential care” OR “long-term care” OR “long term care” OR “disabilit*” OR “disability service” OR “care home” OR “aged care” OR “aged-care” OR “mental health service” OR “mental health centre” OR “mental health facilit*” OR “psychiatric service” OR “psychiatric centre” OR “psychiatric facilit*”  OR “addiction service” OR “addiction centre” OR “addiction facilit*” OR “drug-treatment centre” OR “drug-treatment service” OR “drug-treatment facilit*” OR “drug treatment centre” OR “drug treatment service” OR “drug-treatment facilit*” OR “homecare” OR “home care” OR “domiciliary” OR “primary care” OR “community care” OR “respite care” OR “specialist care” OR “live-in care” OR “live in care” OR “homeless service*” OR “homeless shelter*”

**Intervention**

“regulation” OR “regulator*” OR “inspect*” OR “enforcement” OR “licens*” OR “certification” OR “withdrawal”

**Mechanisms**

“factor*” OR “barrier*” OR “facilitator*” OR “enabler*” OR “determinant*” OR “characteristic*” OR “indicator*” OR “association*” OR “relationship*” OR “cause*” OR “engagement” OR “attitude*”

**Outcome**

“compliance” OR “non-compliance” OR “violat*” OR “deficienc*” OR “sanction*” OR “citation*” OR “failure*” OR “failing*”

**The reviewer’s suggestions here have been valuable and should result in a more comprehensive search strategy from studies on a wide range of settings. The updated search terms are set out below:**

*VII. Are you going to do any citation searching in addition to keyword searching? I think the Mechanism element of CIMO is tricky for this search, and you might miss some research as a result.*

**The protocol makes reference to hand-searching the reference list of included studies in order to identify any further studies for inclusion. However, we agree that the protocol should go further in terms of citation searching as we had intended to complete forward citation, however we did not make it explicit in the previous version. On foot of this comment from the reviewer, we have amended the protocol to now also specify that forward citation searching will be carried out, see addition below.**

**“Forward citation searching will also be carried out to identify other potential material for inclusion.”**

*VIII. Paragraph 3. I did not find the process example particularly illuminating, and wonder if you can find something more obviously related to process. I appreciate that the boundaries between structure and process can be fuzzy, though. The structure and process examples relate these to outcomes. The outcomes example relates outcomes to countries. It might perhaps be better to put this example first, and then to put the others which relate outcome variation to structure and process?*

**The reviewer is justified in querying the applicability of the example used above. We agree and have therefore removed this example and replaced the text with an example that is more closely related to how a process can influence an outcome. See revised example below:**

**“It may be process-related as found in a prospective evaluation of simulated emergency department triage which found a high degree of variability in the processes of triage and measurement of vital signs.”**

**While acknowledging the suggestion that the outcome example could be listed first, we disagree that it should be restructured. We feel it is more appropriate to retain the logical sequence of structure/process/outcome and have, therefore, retained this order in the paragraph.**

*IX. Paragraph 4. "typically fall under" is a bit ambiguous. The structure, process, outcome framework could be applied to any set of standards; it is an empirical question whether governments organise their standards in this way (explicitly or implicitly). This would be strengthened by a supporting reference. If this isn't available, perhaps add an example or two to help back this up?*

**The reviewer is correct in identifying this ambiguity. We agree that is needs revision. We have rephrased this section to remove the ambiguous language. We would also like to draw attention to the examples of regulations framed as structure/process/outcome included in paragraph 5.**

*X. Paragraph 4. "must then comply with regulations...." Strictly speaking, be assessed as complying with regulations, and avoid potential sanctions or other potential negative consequences? Think, for example of reputation (as you highlight later) and "sunshine regulation".*

**We thank the reviewer for clarifying this nuance in terms of what it means to be ‘compliant’ and have changed the terminology used in this section to better reflect the reality, see revised text below:**

**“Regulation is one response to variability in quality: authorities establish a set of norms or standards to benchmark quality and then assess the extent to which organisations and individuals meet these standards. Failure to comply with regulations may lead to sanctions such as intensified surveillance, or even revocation of license to operate (typically through a registration or licensing system).”**

*XI. Column 2, Paragraph 1: CQC as an example of compliant/non-compliant continuum works better with regard to the continuum aspect than the compliant/non-compliant aspect, although CQC assessment does in some ways equate Good with compliant and Requires Improvement with non-compliant. There is also the point that CQC assessments are based on professional judgement and not purely on legal standards, which may complicate things?*

**We thank the reviewer for drawing attention to the manner in which the CQC assesses and rates compliance. While the CQC do not explicitly use the term ‘compliant’ in their rating system, we are of the view that the continuum concept still holds up. As the reviewer points out, the CQC rating system can be mapped quite readily on to more traditional judgments of compliant and not compliant. Therefore, we have retained the example of CQC in this section.**

**We agree with the reviewer’s second point here. We have addressed the point on professional judgment and have included further text to clarify this, see below with new text underlined:**

**“These ratings are determined according to the professional judgment of a CQC inspector and assessed at the level of individual care components; services also receive an overall rating.”**

*XII. Column 2, Paragraph 2: "requires a little more interrogation and evidence gathering". And also a value judgement, although measures of competence etc. might possibly be set out?*

**The reviewer correctly identifies this passage as requiring improvement and agree that it should be amended. We have edited the text to use different examples and be more explicit about what forms of evidence are required by inspectors to make judgments. The revised paragraph is set out below:**

**“Determining the level of compliance with a specific regulation requires varying degrees of effort and evidence-gathering on behalf of an inspector. Evidence can be generated from speaking with residents and staff, reviewing documentation and records, and observing practices as they happen. For example, assessing compliance with a structural requirement, such as a requirement that a person in charge should have “a minimum of 3 years experience in a management or supervisory role in the area of health or social care”** **^10^** **, is a relatively straight-forward task of identifying the appropriate documentation. Judging whether a service has admitted residents in “a competent, equitable, timely, and respectful manner”** **^11^** **, requires the inspector to undertake several tasks: speak with recently-admitted residents or their representatives, review admission records, speak with staff involved in admitting new residents. Assessing compliance in a regulation which specifies an outcome, such as one that seeks to “ensure respect for the personal privacy of each person in care”** **^12^** **, requires the inspector to speak with residents and staff, review documentation and observe care practices on-site.”**

*XIII. Column 2, Paragraph 2: "requires a lot of evidence gathering as well as observation". I don't see how this example is different to admitting in a respectful manner?*

**See response to previous comment XII above.**

*XIV. Also a point perhaps that observation is one way of generating evidence? By evidence gathering perhaps you mean documentary evidence? Could be clearer?*

**See response to previous comment XII above.**
